# In vitro Assessment of Efferocytic Capacity of Human Macrophages Using Flow Cytometry

**DOI:** 10.21769/BioProtoc.4903

**Published:** 2023-12-20

**Authors:** Ana C.G. Salina, Marlon Fortes-Rocha, Larissa D. Cunha

**Affiliations:** 1 Departamento de Biologia Celular e Molecular e Bioagentes Patogênicos, Faculdade de Medicina de Ribeirão Preto, Universidade de São Paulo, Ribeirao Preto, Brazil; 2 Department of Medicine, Division of Infectious Diseases, Vanderbilt University Medical Center, Nashville, TN, USA

**Keywords:** THP-1-derived macrophage, PBMC-derived macrophages, Efferocytosis, Apoptotic cells, Flow cytometry

## Abstract

Clearance of dying cells, named efferocytosis, is a pivotal function of professional phagocytes that impedes the accumulation of cell debris. Efferocytosis can be experimentally assessed by differentially tagging the target cells and professional phagocytes and analyzing by cell imaging or flow cytometry. Here, we describe an assay to evaluate the uptake of apoptotic cells (ACs) by human macrophages in vitro by labeling the different cells with commercially available dyes and analysis by flow cytometry. We detail the methods to prepare and label human macrophages and apoptotic lymphocytes and the in vitro approach to determine AC uptake. This protocol is based on previously published literature and allows for in vitro modeling of the efficiency of AC engulfment during continual efferocytosis process. Also, it can be modified to evaluate the clearance of different cell types by diverse professional phagocytes.

**Graphical overview**


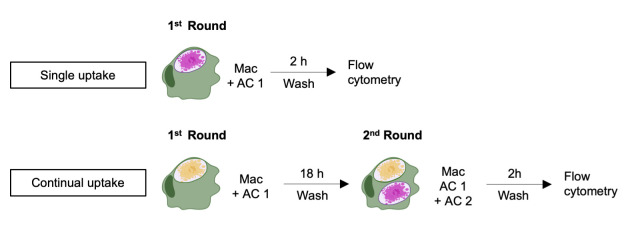


## Background

The uptake of apoptotic cells (ACs) is a signal that modulates macrophage metabolism and gene expression and ultimately shapes their functional programming (Boada-Romero et al., 2020; Schilperoort et al., 2023). Efferocytic activity is coupled to other environmental cues, such as the cytokine milieu and the presence of pathogens, to promote macrophage inflammatory or anti-inflammatory responses and their capacity to induce efficient tissue repair (Torchinsky et al., 2009; Bosurgi et al., 2017; McCubbrey et al., 2022; Salina et al., 2022). Of note, the latter requires efficient continuous uptake of apoptotic cells; thus, one of the consequences of efferocytosis is to promote a positive feedback loop for sequential corpse engulfment (Miyanishi et al., 2007; Park et al., 2011; Yurdagul et al., 2020). The uptake of a single AC or their continual uptake can be assessed by fluorescent cell labeling and analyzed by fluorescence microscopy or flow cytometry (Yurdagul et al., 2020; Gerlach et al., 2021; Salina et al., 2022; Wang et al., 2023). Here, we provide a detailed protocol on how to prepare human macrophages (either from THP-1 cell lines or peripheral blood samples) and evaluate the uptake of apoptotic Jurkat cells by macrophages in vitro using differential fluorescent labeling of cells and data acquisition by flow cytometry. We label each cell population of interest (phagocytes, AC target cells) and show how to identify engulfing populations. This protocol provides a simple approach to probe AC uptake and allows robust quantitative evaluation of continual efferocytic capacity. Of note, variations in cell culture conditions or donor samples may influence macrophage behavior and affect assay results. While designed to assess single and continual efferocytosis in vitro, this protocol could be adapted to facilitate probing AC uptake in challenging models in vivo by pre-labeling target cells and surface staining specific phagocytic populations of interest.

## Materials and reagents

1. 150 cm^2^ cell culture flasks (Corning, catalog number: 430825)
2. Jurkat cells, clone E6-1 (ATCC, catalog number: TIB-152)
3. 5 mL sterile serological pipette (Falcon, Corning, catalog number: 356543)
4. 10 mL sterile serological pipette (Falcon, Corning, catalog number: 357551)
5. 25 mL sterile serological pipette (Falcon, Corning, catalog number: 357525)
6. 50 mL sterile polypropylene conical tube (Corning, catalog number: SCT-50ML-25-S)
7. 24-well tissue culture plate (Corning, Costar, catalog number: 3524)
8. 5 mL syringe (BD Bioscience, catalog number: 990317)
9. Hypodermic needle 23 G × 1 (BD Bioscience, catalog number: 300388)
10. Disposable Pasteur pipette
11. 28 mm diameter syringe filter, 0.2 µm pore PES membrane (Corning, catalog number: 431229)
12. Cell scraper (Corning, Falcon, catalog number: 353085)
13. 5 mL non-sterile round-bottom polystyrene test tube (FACS tube) (Falcon, Corning, catalog number: 352008)
14. THP-1 cells (ATCC, catalog number: TIB-202)
15. Roswell Park Memorial Institute (RPMI) 1640 medium (Thermo Fisher Scientific, Gibco, catalog number: 22400-089)
16. Fetal bovine serum (FBS) (Thermo Fisher Scientific, Gibco, catalog number: 12657-029)
17. GlutaMAX^TM^ supplement (Thermo Fisher Scientific, Gibco, catalog number: 35050-061)
18. Penicillin-streptomycin (10,000 U/mL) (Thermo Fisher Scientific, Gibco, catalog number: 15140-122)
19. Phosphate buffer saline (PBS) (Thermo Fisher Scientific, Gibco, catalog number: 10010-023)
20. CellTrace^TM^ Far Red (Thermo Fisher Scientific, Invitrogen, catalog number: C34572)
21. pHrodo^TM^ Red AM intracellular pH indicator dye (Thermo Fisher Scientific, Invitrogen, catalog number: P35372)
22. Tissue culture dish (100 mm × 20 mm) (Corning, catalog number: 430591)
23. Phorbol 12-myristate 13-acetate (PMA) (InvivoGen, catalog number: Tlrl-pma)
24. Leukocyte reduction system (LRS) cone
25. Histopaque^®^-1077 (Sigma-Aldrich, catalog number: 10771-500ML)
26. Ammonium chloride (NH_4_Cl) (CAS 12125-02-9) (Mallinckrodt, catalog number: 3348)
27. Potassium bicarbonate (KHCO_3_) (CAS 298-14-6) (Sigma-Aldrich, catalog number: 237205)
28. Ethylenediaminetetraacetic acid (EDTA) (CAS 60-00-4) (Amresco, catalog number: 0322-500G)
29. Paraformaldehyde 20% aqueous solution EM grade (Electron Microscopy Sciences, catalog number: 15713)
30. CellTrace^TM^ CFSE (Invitrogen, Thermo Fisher Scientific, catalog number: C34554)
31. CellTrace^TM^ Violet (Invitrogen, Thermo Fisher Scientific, catalog number: C34571)
32. Human serum from human male AB plasma (human serum), USA origin, sterile-filtered (Sigma-Aldrich, catalog number: H4522-100ml)
33. Zombie NIR Fixable Viability (BioLegend, catalog number: 423106)
34. ACK lysis buffer (see Recipes)

**Recipes**

1. **ACK lysis Buffer**
  0.15 M NH_4_Cl
  10 mM KHCO_3_
  0.1 mM EDTA
  Solvent: deionized H_2_O
  - Adjust the pH solution to 7.3.
  - Filter the solution using a 0.2 μm sterile filter and keep it at 4 °C.

## Equipment

1. Hemostatic forceps
2. Surgical scissor
3. Centrifuge (Thermo Scientific, Heraeus Megafuge 40R, catalog number: 50119920)
4. UV Crosslinker (Fisher Scientific, model: 234100, catalog number: 13-245-221)
5. Automated cell counter (Life Technologies, model: Countless II FL, catalog number: AMQAF1000) or cell counting chamber
6. Flow cytometer (BD Biosciences, FACS Verse, catalog number: 651153)

## Software

1. FlowJo v10.8.0 (FlowJo, LLC, www.flowjo.com)
2. Prism 8 (GraphPad Software, Inc., www.graphpad.com)

## Procedure

*Note: Suggested conditions are sufficient to prepare one 24-well tissue culture plate of macrophages and AC for the efferocytosis assay, including flow cytometer compensation controls and fluorescence-minus-one controls for cell gating at Data analysis.*

- **THP-1-derived macrophage differentiation**
*Note: THP-1 cells are maintained in 175 cm^2^ cell culture flasks in RPMI culture medium supplemented with 10% of FBS, 1% GlutaMAX^TM^ supplement, and 1% penicillin-streptomycin medium (RPMIc), at 37 °C and 5% CO_2_ atmosphere. The cell culture is split, and the medium is replenished every other day. Whenever mentioned below, cell transferring and resuspension were performed using disposable serological pipettes. All media and buffers were pre-warmed prior to use.*
  1. Collect the cell suspension from the flask, transfer to a 50 mL conical tube, and pellet them by centrifugation (400× *g* for 5 min at room temperature).
  2. Dump the medium, resuspend the pelleted cells in 5 mL of PBS, and count them.
    - Transfer 2.4 × 10^7^ THP-1 cells to a clear 50 mL conical tube (Tube A) and centrifuge them.
    - Transfer at least 4.0 × 10^6^ THP-1 cells to another clear 50 mL tube (Tube B). Centrifuge them, dump the media, and resuspend the cell pellet in fresh RPMIc at 4.0 × 10^6^ cells/mL. These cells will be spared to prepare compensation controls (unstained and viability dye single-color controls) and should be kept at 37 °C and 5% CO_2_ until seeding.
  3. Dump the medium in tube A and resuspend the THP-1 cells in 2.4 mL of PBS (1.0 × 10^7^ cells/mL).
  4. Proceed to cell labeling with CellTrace^TM^ Violet or CFSE (working solution concentration: 5 μM) following manufacturer's instructions (Note 1).
    - Add the labeling reagent at 1:1,000 to obtain adequate working concentration, mix by flicking the tube, and incubate for 20 min protected from light in a 37 °C water bath.
    - Add five times the original staining volume of fresh pre-warmed cell media to the tube and incubate for 5 min at 37 °C.
    - Centrifuge the tube (400× *g* for 5 min).
    - Dump the medium and rinse the cell pellet with 10 mL of fresh pre-warmed cell media.
  5. Following the washout step, resuspend the THP-1 cell pellet in 6 mL of fresh RPMIc (4.0 × 10^6^ cells/mL).
  6. Seed 250 µL (1.0 × 10^6^ cells) of the cell suspension in tubes A and B per well of a 24-well cell culture plate.
  7. Prepare a solution of PMA at 100 ng/mL using RPMIc as diluent.
  8. Add 250 µL of PMA solution to each well (final volume 500 μL, final concentration 50 ng/mL).
  9. Incubate the plate for 24 h at 37 °C and 5% CO_2_.
  10. Aspirate all the medium, replenish with 500 μL of fresh RPMIc, and incubate the plate for another 24 h at 37 °C and 5% CO_2_.
  11. Macrophages are ready for efferocytosis assay.
- **PBMC-derived macrophage differentiation**
*Note: This protocol uses peripheral blood mononuclear cells (PBMC) purified from leukocyte reduction system (LRS) cones obtained from the apheresis of donated blood samples. Monocytes are sorted by adherence to generate primary macrophages.*
  1. Collect the LSR cone and preserve it at 4 °C until use.
  2. Lock one of the LRS cone accesses with hemostatic forceps.
  3. Use a sterile surgical scissor to cut the opposite LRS cone access.
  4. Insert a 23 G hypodermic needle attached to a 5 mL syringe into the cone, collect the cells, and transfer them to a 50 mL conical tube.
  5. Transfer up to 2.5 mL of the collected sample to a 50 mL conical tube. Split the sample into two conical tubes if necessary.
  6. Prepare a cell suspension by adding 35 mL of cold PBS to the sample and thoroughly mix by up-and-down pipetting.
  7. Transfer 13 mL of Histopaque^®^-1077 solution to a clear 50 mL conical tube.
  8. Overlay the cell suspension onto the top of Histopaque^®^-1077 solution without mixing by carefully dropping the cell suspension on the tube wall using a sterile disposable Pasteur pipette.
  9. Set up the centrifuge to medium acceleration and no break. Centrifuge the gradient at 640× *g* for 30 min at 4 °C. If successful, the formation of a gradient with four separate layers should be clear (Figure 1). Carefully manipulate the tube to avoid mixing them up.
    **Figure 1.**
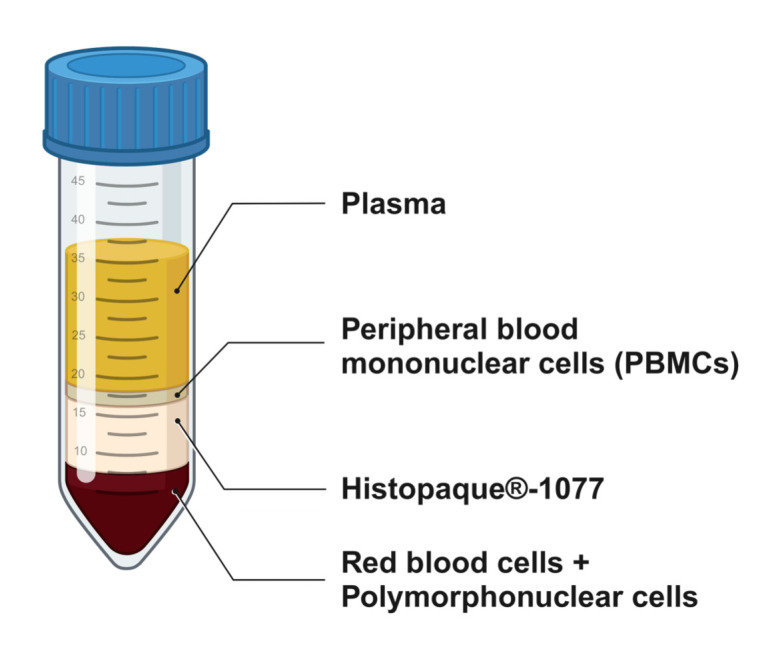
Schematics of the gradient obtained by centrifugation to isolate peripheral blood mononuclear cells (PBMCs)
  10. Using a disposable Pasteur pipette, discard the top layer (remaining plasma and thrombocytes).
  11. Using a new disposable Pasteur pipette, collect the intermediate layer containing PBMCs and transfer to a clear 50 mL conical tube.
  12. Resuspend the PBMC layer in 25 mL of cold PBS and centrifuge the cells (400× *g* for 5 min).
  13. Dump the PBS and resuspend the PBMCs in 5 mL of cold ACK lysis buffer (see Recipes).
  14. Incubate the PBMC solution on ice for 5 min.
  15. Add 25 mL of PBS to the tube, mix by pipetting, and centrifuge the cells.
  16. Dump the PBS, resuspend the PBMCs in 5 mL of pre-warmed PBS, and count the PBMCs.
    - Transfer 2.4 × 10^8^ PMBCs to a clear 50 mL conical tube (Tube A) and centrifuge them.
    - Transfer 4.0 × 10^7^ PBMCs to a clear 50 mL conical tube (Tube B) for compensation controls as described in step A2b. Centrifuge the tube, dump the PBS, and resuspend the PBMC pellet in 2 mL of fresh pre-warmed RPMI without serum (2.0 × 10^7^ cells/mL). The cells should be kept at 37 °C and 5% CO_2_ until seeding.
  17. Dump the medium in tube A and resuspend the PBMC in 2.4 mL (10^8^ cells/mL) of pre-warmed PBS.
  18. Proceed to cell labeling with CellTrace^TM^ Violet or CFSE (working solution concentration: 5 μM) following steps A4a–A4d.
  19. Following the washout step, resuspend the PBMC pellet in 12 mL of fresh pre-warmed RPMI without serum (2.0 × 10^7^ cells/mL).
  20. Seed 500 µL (1.0 × 10^7^ cells) of the PBMC suspension in tubes A and B per well of a 24-well cell culture plate and gently swirl the plate to evenly distribute the cells. Spare 2–3 of the wells seeded with labeled PBMCs (from tube A) to confirm the amount of differentiated macrophages immediately before proceeding to the efferocytosis assay.
  21. Incubate the plate for 1 h at 37 °C and 5% CO_2_.
  22. Thoroughly rinse each well thrice with pre-warmed PBS to remove unattached leucocytes. Monocyte sorting by adherence should give on average 1.0 × 10^6 ^cells/well.
  23. Aspirate all the medium and replenish with 1 mL of pre-warmed RPMI supplemented with 10% of human serum (RPMIh) per well.
  24. Incubate the plate for 72 h at 37 °C and 5% CO_2_.
  25. Add 1 mL of fresh pre-warmed RPMIh per well and incubate the plate for 72 h at 37 °C and 5% CO_2_.
  26. Lift the macrophages of 2–3 wells using a cell scraper and count cells to confirm cell confluence (Note 2).
  27. Macrophages are ready for efferocytosis assay.
- **Generation of stained apoptotic Jurkat cells**
*Note: Jurkat cells are maintained in 175 cm^2^ cell culture flasks in RPMIc at 37 °C and 5% CO_2_. The medium is replaced every other day. Whenever mentioned below, cell transferring and resuspension were performed using disposable serological pipettes. All media and buffers were pre-warmed prior to use.*
  1. Collect the cell suspension from the flask, transfer to a 50 mL conical tube, and pellet them by centrifugation (400× *g* for 5 min at room temperature).
  2. Dump the medium, resuspend the pelleted cells in 5 mL of fresh RPMIc, and count them.
  3. Transfer 3.0 × 10^7^ Jurkat cells to a 50 mL conical tube and centrifuge them.
  4. Dump the medium and resuspend the pelleted cells in 3 mL of fresh RPMIc.
  5. Transfer the cells to a tissue culture dish (100 mm × 20 mm).
  6. Using the UV crosslinker, irradiate the tissue culture dish at 50 mJ/cm^2^ (Notes 3 and 4).
  7. Collect the irradiated cells and transfer them to a clear 50 mL conical tube (Tube A).
  8. Rinse the tissue culture dish to collect remaining cells with 9 mL of PBS and transfer the suspension to Tube A. Transfer 2.0 mL of the cell suspension (5.0 × 10^6^ cells) from Tube A to another clear 50 mL tube (Tube B). Centrifuge them, dump the media, and resuspend the pelleted cells in 10 mL of fresh RPMIc. These cells will be spared to prepare experimental control tubes FMO AC 1 and FMO AC 2 (Figure 1).
  9. Centrifuge the remaining cells in tube A, discard the medium, and resuspend the cells in 3 mL of PBS (1.0 × 10^7^ cell/ mL).
  10. Proceed to cell labeling with CellTrace^TM^ Far Red (working solution concentration: 1 μM) or pHrodo^TM^ Red (working solution concentration: 5 nM), following steps A4a–A4d (Notes 5 and 6).
  11. Following the washout step, resuspend labeled ACs in 10 mL of fresh RPMIc.
  12. Transfer the cell suspensions in Tube A and B to separate tissue culture dishes.
  13. Incubate them for 4 h at 37 °C and 5% CO_2_ to allow apoptosis to proceed.
  14. Collect the ACs and transfer them to 50 mL conical tubes.
  15. Rinse the tissue culture dish to collect remaining cells with 10 mL of pre-warmed PBS and transfer the suspension to Tube A.
  16. Centrifuge the AC tubes, discard the medium, resuspend the cells in 5 mL of RPMIc or RPMIh (if using THP1- or PBMC-derived macrophages assays, respectively), and count them.
  17. Adjust the cell concentration to 5.0 × 10^6^ AC/mL with RPMIc or RPMIh and proceed to efferocytosis assay.
- **Efferocytosis assay and sample preparation for data acquisition**
  1. Rinse the plate of CFSE-labeled macrophages with 500 μL of pre-warmed PBS per well.
  2. Transfer 200 µL of the labeled AC 1 preparation in tube A to each well of the experimental group for single uptake or first round of continual uptake. This should give a 1:1 macrophage to AC ratio (cell ratio may vary according to experimental settings).
  3. Transfer 200 µL of the unlabeled AC 1 preparation in tube B to each well of the FMO AC 1 control group (Figure 1).
  4. Gently swirl the plate to evenly distribute the ACs.
  5. Incubate the plate for 2 h at 37 °C and 5% CO_2_.
  - If performing single uptake, proceed to step D11.
  - If performing continual efferocytosis assay, incubate the plate for 18 h instead at 37 °C and 5% CO_2_. Proceed to step D6 (Note 7).
    1. Remove the cell medium in each well and rinse the plate thrice using pre-warmed 500 μL of PBS per well.
    2. Transfer 200 µL of the labeled AC 2 preparation in tube A to each well of the experimental group fed with labeled AC 1.
    3. Transfer 200 µL of the unlabeled AC 2 preparation in tube B to each well of the FMO AC 2 control group fed with labeled AC 1 (Figure 1).
    4. Gently swirl the plate to evenly distribute the ACs.
    5. Incubate the plate for 2 h at 37 °C and 5% CO_2_.
    6. Rinse the plate thrice with pre-warmed 500 μL of PBS per well.
    7. Add 200 μL of PBS to each well.
    8. Lift the cells using a cell scraper. Rinse or replace the cell scraper between each group.
    9. Transfer the samples to FACS tubes.
    10. Prepare a solution by diluting Zombie NIR in PBS with enough volume for all samples (final dilution 1:400, final staining volume per tube: 50 μL).
    11. Centrifuge the samples (400× *g* for 5 min).
    12. Resuspend the pellets in 50 μL of Zombie NIR solution.
    13. Incubate the samples on ice for 10 min in the dark.
    14. Centrifuge the samples.
    15. Dump the solution and rinse the samples twice with 500 μL of PBS per tube. If necessary, resuspend the cells in 250 μL of a PBS solution with 2% paraformaldehyde and incubate for 15 min on ice for cell fixation.
    16. Resuspend the pellet in 250 μL of PBS and proceed to data acquisition with a flow cytometer.

## Data analysis

*Note: Gating strategy to quantify AC uptake in single and continual efferocytosis is shown in Figure 1. We recommend acquiring at least 50,000 total events*.

1. Export acquired data to .fcs file format and analyze using the software FlowJo.
2. Apply morphological criteria to gate the macrophage cluster and exclude debris based on forward (FSC) and side scatter (SSC) profiles.
3. Exclude doublets creating a gate through the diagonal area on FSC-A vs. FSC-H plot.
4. Exclude dead cells based on the staining for Zombie NIR viability dye.
5. Select the macrophages based on the positive staining for CFSE.
6. In the CFSE^+^ macrophage subpopulation, create a gate for the first round of AC uptake (AC1) using FMO AC1 as the reference tube. The percentage of positive cells in this gate represents the rate of the single uptake or first round of the continual uptake.
7. If performing continual uptake, create a gate for the second AC uptake in the CFSE^+^ AC-pHrodo^+^ macrophage subpopulation, using FMO AC2 as the reference tube. The percentage of positive cells in this gate represents the percentage of labeled macrophages that engulfed AC1 and were also efficient in the uptake of a second AC.

## Validation of protocol

**Figure 2.**
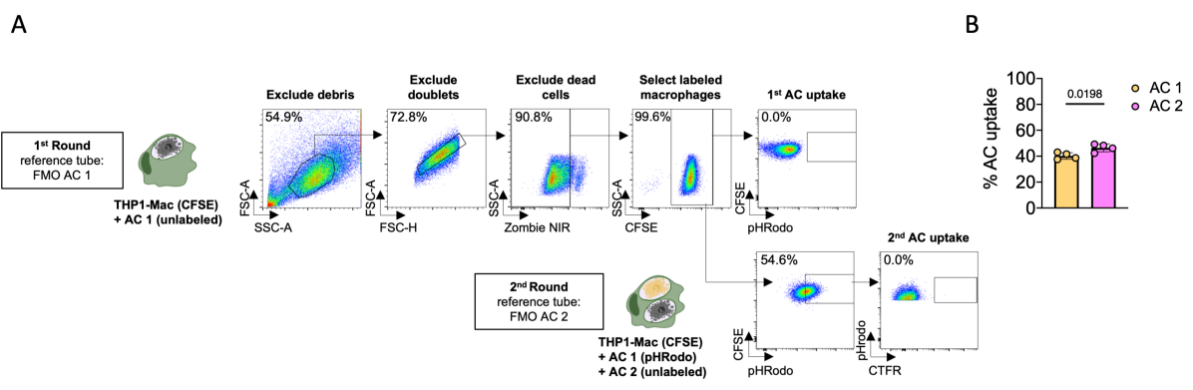
Flow cytometric analysis to determine the uptake of apoptotic cells by the macrophages. THP-1-derived macrophages (CTV-labeled) were incubated with UV-irradiated apoptotic Jurkat cells for 18 h (first round, pHRodo-labeled AC1), and subsequently incubated with a second batch of apoptotic Jurkat cells for 2 h (second round, CTFR-labeled AC2), following the schematics described on the Graphic Abstract. (A) Representative flow plot of gating strategy using FMO controls. Macrophages are initially selected, and debris are excluded based on morphology profile on FSC-A vs. SSC-A plot. Next, doublet exclusion is performed on FSC-A vs. FSC-H plot. Next, dead cells were excluded based on staining for viability dye (Zombie-NIR). Next, labeled macrophages (CFSE^+^) were selected. Gating for labeled macrophages that uptake AC on the single uptake or the first round (AC1) is performed using FMO AC1 sample as reference control. Gating for labeled macrophages that uptake AC on the second round (AC2) is performed using FMO AC2. (B) Percentage of macrophages with internalized AC1 and AC2. Boxes represent the mean of four biological replicates and error bars are ± S.E.M. Each biological replicate is shown as a circle. Significance was calculated by Student’s *t*-test.

An example of the percentage of apoptotic cell uptake in the first (AC1) and second (AC2) rounds of efferocytosis by THP-1 cells following the steps described in the Procedure and Data analysis section is shown in Figure 2B.

This protocol or parts of it has been used and validated in the following research article:

Salina et al. (2022). Efferocytosis of SARS-CoV-2-infected dying cells impairs macrophage anti-inflammatory functions and clearance of apoptotic cells. *eLife*, 11, e74443.

Data of single and continual uptake of AC by PBMC and THP-1-derived macrophages is presented in Figure 1 and supplement Figure 1 and Figure 4 of the aforementioned article.

## Notes

1. It is possible to use cell surface staining following the efferocytosis assay to label the macrophages instead of using fluorescent probes. However, in our experience and as reported by others (Forrester et al., 2018), we found that THP-1-derived macrophages may express low levels of common surface markers used for phenotyping.
2. We recommend confirming macrophage differentiation by flow cytometry phenotyping.
3. Induction of apoptosis by UV irradiation may need to be adjusted according to different equipment.
4. We recommend the evaluation of the efficiency of apoptosis induction using standard apoptosis assay using flow cytometry (i.e., using fluorescent annexin V and viability dye co-staining). If inducing cell death with other methodologies (i.e., pharmacological drugs or by genetic manipulation), labeling may need to be performed prior to induction.
5. The amount of Jurkat cells for labeling can be scaled but avoid altering cell concentration. Ideal cell concentration for optimum labeling may vary for different cell types used to prepare the AC.
6. There is contradicting data on the literature about CFSE leakage from apoptotic cells. For that reason, we do not recommend its use to label the ACs. Other commercially available fluorescent probes may be used to label target ACs and assess their uptake by flow cytometry, such as CypHer5E, DAPI, and PKH26.
7. Incubation time for the first round of continual uptake may vary according to experimental settings. Labeling with pHrodo^TM^ Red is associated with phagolysosome acidification and its specificity should be taken into consideration for ideal experimental design.
